# Inhibitory deficits and symptoms of attention‐deficit hyperactivity disorder: How are they related to effortful control?

**DOI:** 10.1111/bjdp.12432

**Published:** 2022-09-20

**Authors:** Katarzyna Kostyrka‐Allchorne, Sam V. Wass, Hodo Yusuf, Vidya Rao, Chloé Bertini, Edmund J. S. Sonuga‐Barke

**Affiliations:** ^1^ Department of Child and Adolescent Psychiatry Institute of Psychiatry, Psychology & Neuroscience, King's College London London UK; ^2^ School of Psychology University of East London London UK; ^3^ Department of Child & Adolescent Psychiatry Aarhus University Aarhus Denmark

**Keywords:** ADHD, conduct problems, effortful control, inhibitory control, temperament

## Abstract

Separate studies with clinical and community‐based samples have identified an association between symptoms of attention‐deficit hyperactivity disorder (ADHD) and inhibitory control deficits and ADHD and weak effortful control. We tested whether differences in effortful control explained the associations between ADHD symptoms and inhibitory control deficits, controlling for conduct problems. In a community sample, parents rated ADHD symptoms, conduct problems, effortful control, surgency and negative affect in 77 4‐7‐year‐olds (47 girls), who performed an inhibitory control task. ADHD symptoms, deficient inhibitory control and low effortful control were correlated. Controlling for conduct problems, path analysis showed the ADHD symptoms – inhibitory control link was mediated statistically by effortful control. This focuses attention on cognitive‐energetic factors associated with ADHD‐related executive deficits.

**Statement of contribution**

**Existing knowledge:**

- Inhibitory control (IC) has been singled out as primary executive function (EF) affected in ADHD.
- ADHD symptom are linked to low effortful control, a temperament factor that conceptually overlaps with EF.
- Conduct problems (CP) that often cooccur with ADHD, are also linked to temperament differences.

**New knowledge:**

- After controlling for CP, ADHD symptoms‐IC link was fully statistically mediated by effortful control.
- In contrast, CP were directly associated with poor IC.
- The nature of IC deficits may be unique to the type of psychopathology.

## BACKGROUND

Attention‐deficit hyperactivity disorder (ADHD) is a neurodevelopmental condition characterized by persistent and age‐inappropriate hyperactivity, impulsivity and inattention, which disrupt daily functioning and may interfere with healthy development. ADHD is characterized by heterogeneity in neurocognitive deficits and a diverse range of behavioural symptoms, which vary by sex and developmental stage and are often accompanied by emotional difficulties (Posner et al., 2020). Substantial evidence suggests that ADHD is best conceptualized as a continuum of symptoms that may vary across development and context rather than a discrete diagnostic category (e.g., Larsson et al., 2012; Lubke et al., 2009; Sonuga‐Barke et al., 2002). Therefore, the present research included a community sample of children with a range of ADHD symptoms.

The evidence from over three decades of research points to deficits in executive function as one of the key neuropsychological correlates of ADHD (Bayliss & Roodenrys, 2000; Craig et al., 2016; Pennington & Ozonoff, 1996; Willcutt et al., 2005). Executive function encompasses higher‐order cognitive processes that underpin goal‐oriented behaviour, for example, working memory, planning and organization, mental flexibility and inhibitory control (Diamond, 2013). Individual differences in childhood executive function are an important correlate of a range of outcomes, including academic achievement (Ahmed et al., 2019; Willoughby et al., 2019), emotional and behavioural adjustment (Cassidy, 2016) and physical health (Reinert et al., 2013; Stautz et al., 2016).

Of the core executive function domains affected in ADHD, inhibitory control deficits have been singled out as primary (Barkley, 1997). Inhibitory control refers to the ability to stop an inappropriate but dominant response (Simpson & Carroll, 2019) and "makes it possible for us to change and for us to choose how we react and how we behave rather than being unthinking creatures of habit" (Diamond, 2013, p. 137). Crucially, inhibitory control is activated when conflict is triggered by a mismatch between someone's goal and their pre‐disposition either to engage in routine behaviour or to give a pre‐prepared response (Inzlicht et al., 2015). Evidence of deficient inhibitory control in ADHD have been found across different tasks in studies using both case–control designs (Lansbergen et al., 2007; Lipszyc & Schachar, 2010; Mullane et al., 2009; Oosterlaan et al., 1998), as well as dimensional approaches in community samples (Berlin & Bohlin, 2002; Thorell & Wåhlstedt, 2006; Tillman et al., 2007).

More broadly, executive function is thought to be related to constitutional differences in regulation and reactivity captured by temperament (Bates et al., 2009). Temperament refers to a moderately stable set of early appearing traits that reflect both the extent to which individuals respond to their environment (i.e., reactivity) and their ability to modulate and control these responses (i.e., regulation; Rothbart, 2004). In the taxonomy proposed by Rothbart and colleagues, temperament is organized into three broad factors of effortful control, negative affect and surgency (Putnam et al., 2001; Putnam & Rothbart, 2006; Rothbart et al., 2001).

Individuals with ADHD have been shown to score low on effortful control (De Pauw & Mervielde, 2011; Krieger et al., 2019; Martel et al., 2012; Rutter & Arnett, 2021; Samyn et al., 2011). Reduced effortful control is also associated with ADHD symptoms in non‐clinical samples (Kerner auch Koerner et al., 2018; Martel et al., 2014). Effortful control comprises attention shifting, attention focusing, inhibitory control and perceptual sensitivity (Rothbart et al., 2001). Effortful control is domain‐general; it serves to regulate emotions, as well as attention and action (Nigg, 2017). Crucially, there is a substantial conceptual overlap between effortful control and executive function (Bridgett et al., 2013; Gagne et al., 2021; Zhou et al., 2012), with attentional and inhibitory processes providing a common platform for regulatory behaviours (Liew, 2012). While empirical evidence on the degree of overlap between effortful control and executive function in developmental samples is limited, recent data showed that these two constructs are moderately correlated and that inhibitory and cognitive control serves as the common regulatory dimensions (Kim‐Spoon et al., 2019).

Here, we explore the inter‐relationship between symptoms of ADHD, inhibitory control and effortful control. Specifically, we test the hypothesis that the ADHD‐inhibitory control deficits link is underpinned by shared temperamental differences in effortful control. To date, only one study examined the complex associations between temperament, executive function and ADHD symptoms (Krieger et al., 2019). The authors examined the role of the key temperament factors in mediating the associations between adolescent ADHD symptoms and executive functions (including inhibitory control). Bivariate correlations showed that ADHD symptoms were negatively associated with inhibitory and effortful control. Further, the structural equation model, which combined inhibitory control, working memory and planning into one executive function variable, showed that effortful control but not negative affect or surgency was a significant mediator of the association between executive function and ADHD symptoms.

In this study, we built on previous work by exploring the specificity of the ADHD symptoms – inhibitory control – effortful control relationship in two ways. First, we tested the hypothesis that the ADHD symptoms – inhibitory control deficit link was specifically underpinned by temperamental differences in effortful control by additionally comparing it to two other temperament factors – negative affect and surgency. Negative affect encompasses lower‐order traits of anger, sadness, fear, physical discomfort and recovery from distress (Rothbart et al., 2001) and it is thought to be the hallmark of reactivity. Childhood negative affect has been linked to ADHD both cross‐sectionally (Brammer & Lee, 2013; De Pauw & Mervielde, 2011; Healey et al., 2011; Martel et al., 2014) and longitudinally (Abulizi et al., 2017; Kostyrka‐Allchorne et al., 2020). In particular, anger and frustration have been singled out as the key temperamental correlates of ADHD (Miller et al., 2019; Rabinovitz et al., 2016). Surgency comprises of high‐intensity pleasure, positive anticipation, impulsivity/sociability and activity level, though the overall evidence for the associations between surgency and ADHD symptoms is tenuous. For example, De Pauw and Mervielde (2011) found no difference in parent‐rated surgency between typically developing and ADHD groups of 6‐ to 14‐years‐old children. In contrast, surgency was associated with both inattention and hyperactivity of 3‐ to 6‐year‐old children, but only at the time of baseline measurement and was not predictive of 1 year course of ADHD symptoms (Martel et al., 2014).

Second, we tested the clinical specificity of the relationships by controlling for conduct problems, which often co‐occur with ADHD and have been linked to temperamental differences. Conduct problems manifest in a persistent pattern of antisocial behaviour that includes oppositionality, defiance, lying, stealing and bullying (American Psychiatric Association, 2013). Prior research showed that temperamental factors in childhood, such as increased negative affect (Martel & Nigg, 2006; Rutter & Arnett, 2021; Sanson et al., 2009) and reduced effortful control (Oldehinkel et al., 2004) are associated with broadly defined externalizing difficulties, including conduct disorder.

The few studies that have examined the associations between ADHD and different temperamental factors, and ADHD and different aspects of self‐regulation (i.e., cognitive and emotional) while also accounting for conduct disorder, showed that these psychopathologies were characterized by specific temperamental and regulatory profiles (Graziano et al., 2013; Martel et al., 2012; Sjöwall et al., 2015). Specifically, conduct problems were associated with more negative affect, whereas ADHD with low effortful control and higher surgency. Therefore, accounting for the co‐occurrence of ADHD symptoms and conduct problems would provide a clearer picture of the respective roles of temperamental factors and self‐regulation in relation to these two psychopathologies, which are so often overlapping.

In sum, the present study aimed to explore the inter‐relationship between ADHD‐related deficits in inhibitory control and effortful control in children aged 4‐ to 7 years. We focused on this developmental period, as it covers the transition from a play‐oriented environment, such as pre‐school or home, to primary school where there is a greater need to inhibit irrelevant or inappropriate behaviour to learn and develop social relations. ADHD symptoms and effortful control were measured with parental responses on the Strength and Difficulties Questionnaire (SDQ; Goodman, 1997) and Children's Behaviour Questionnaire (CBQ; Putnam & Rothbart, 2006), respectively. Inhibitory control was measured with the age‐appropriate version of the AX‐Continuous Performance Task (AX‐CPT). This task has been successfully used in a range of populations, including young children (Chatham et al., 2009; Gonthier et al., 2019; Lucenet & Blaye, 2014; Traut et al., 2021).

In the AX‐CPT task, children are required to make a target response on 70% of the trials and so the requirement to make a rare non‐target response on the remaining 30% of the trials creates high inhibitory demands. Studies that measured children's neural activity during this task, showed that the amplitude of N2, the event‐related potential component proposed to reflect processes involved in inhibition, was significantly larger in the trials that created higher inhibitory demands (Overtoom et al., 1998; Spronk et al., 2008). This is typically recorded when there is a conflict between the dominant response and the response required in rare trials (Spronk et al., 2008) and has been observed across other inhibitory control paradigms, such as go/no‐go tasks (for discussion see Hoyniak, 2017).

The AX‐CPT task is typically used to empirically distinguish two types of inhibitory control: proactive and reactive. *Proactive control* involves maintaining task‐relevant information and advanced goal‐directed response preparation. Conversely, *reactive control* is engaged to correct conflict between a pre‐prepared response and the goal have been detected (Braver, 2012). Interestingly, prior research has consistently shown that children with ADHD diagnoses or with elevated ADHD symptoms have impaired reactive but not proactive control (e.g., Kenemans, 2015; Suarez et al., 2021; van Hulst et al., 2018) and reduced reactive inhibition has been proposed as one of the core deficits that distinguishes ADHD from other neurodevelopmental disorders (Kenemans, 2015). Finally, the shift from less mature reactive control to more efficient proactive control is gradual and most likely begins around the age of 5 to 6 years (Blackwell & Munakata, 2014; Gonthier et al., 2019; Lucenet & Blaye, 2014), which broadly aligns with the age of the children in the present study.

Prior literature provides consistent evidence that ADHD is characterized by deficits in inhibitory control and also points to low effortful control as one of the key individual differences related to ADHD symptoms. Considering the conceptual and empirical overlap between these two constructs, our hypothesis predicted that the ADHD‐inhibitory control deficit link was statistically mediated by temperamental differences in effortful control. ADHD symptoms often co‐occur with conduct problems and have been also linked to other temperamental differences. Therefore, we also tested the specificity of the associations between temperament, inhibitory control and childhood psychopathology symptoms, by including parental reports of negative affect, surgency (measured with the CBQ; Putnam & Rothbart, 2006) and controlling for conduct problems measured with the SDQ (Goodman, 1997).

## MATERIALS AND METHODS

### Participants

A convenience sample of 77 children (30 boys) aged from four to 7 years (*M* = 5.5 years, *SD* = 1.0 years) and their parents were recruited from an urban location in the United Kingdom. A further eight children took part but were excluded from the study (two did not want to complete the inhibitory control task, three did not understand the task, parents of three children failed to complete the questionnaires). Parents and children were recruited from the database of families who expressed interest in taking part in developmental research and via social media advertisements. Although participating families represented diverse ethnic communities, the sample was particularly well‐educated, with 78% reporting having a university degree (Table 1).

**TABLE 1:** Characteristics of the child and parent sample.

	%	*N*
Gender of child		
Male	39.0	30
Female	61.0	47
Ethnic group of children		
White	35.1	27
Black	14.3	11
Asian	18.2	14
Mixed	28.6	22
Other	2.6	2
Undisclosed	1.3	1
Highest level of parent's education		
GCSE	3.9	3
A‐levels	5.2	4
Vocational qualifications	7.8	6
Undergraduate degree	31.2	24
Post‐graduate degree	46.8	36
Undisclosed	5.2	4

The project received ethical approval from The University of East London Research Ethics Committee. Before the study began, the researchers provided parents with information about the project and the experimental procedure and obtained written consent. Children provided assent before taking part. Children were tested individually in the laboratory, and each session took approximately 1 hour. As thanks for taking part, each child received £5 and a certificate.

#### Inhibitory control task and materials

To measure inhibitory control, we used a modified version of the AX‐CPT paradigm (Chatham et al., 2009). In the AX‐CPT task, children are required to make a frequent target response (70% of the trials) every time a probe ‘X’ is directly preceded by a cue ‘A’ (‘AX’ combination). In between these trials, other cue‐probe combinations appear (e.g., ‘A’ followed by ‘Y’, ‘X’ preceded by ‘B’ and ‘B’ followed by ‘Y’), which require a rare non‐target response (10% for each combination). Trials AY and BX both include a stimulus that is included in the target AX combination and so are critical to assessing the mode of control. The outcome variables were two d‐prime indices reflecting inhibitory control derived from signal detection theory (Stanislaw & Todorov, 1999).

The task was programmed in E‐Prime 2.0. Children were required to match a character (Sponge Bob or Blue) with a fruit (watermelon or orange) according to the rules provided by the experimenter. For example, ‘Sponge Bob likes watermelon. When you see watermelon press a smiley face on this box’. ‘Sponge Bob does not like oranges. When you see an orange, press an unhappy face on this box’. Before the task commenced, the experimenter explained the rules using A4 cards showing the correct pairings and asked the children to repeat the instructions and show which buttons to press. After receiving instructions, the children completed 12 practice trials. Children made responses using a response box, where two buttons were marked with stickers: a yellow happy face to indicate ‘like’ responses and a red unhappy face to indicate ‘dislike’ responses. The blocks of task trials were interspersed with periods of rest lasting 2 min, during which children watched a cartoon.

**FIGURE 1:**
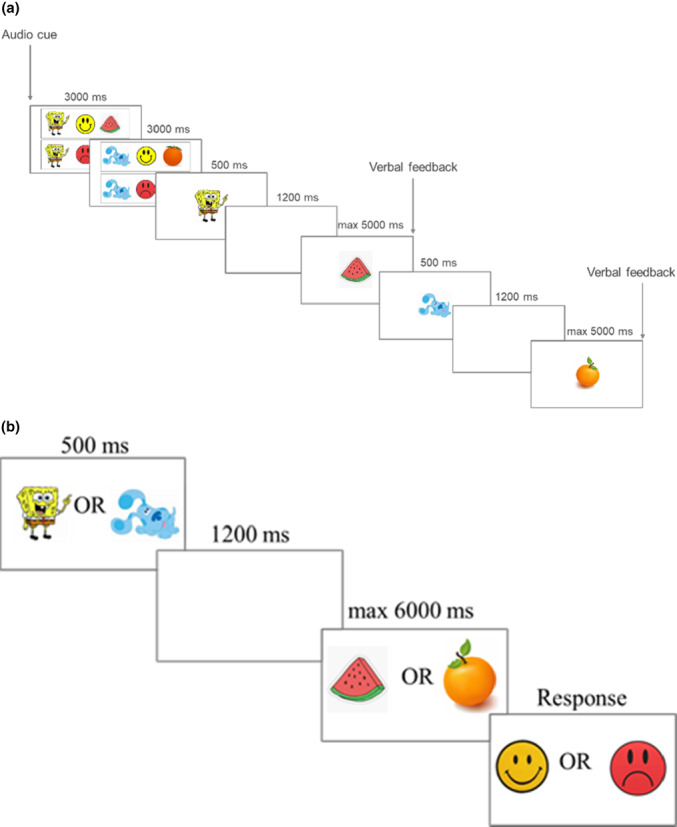
(a) Schematic representation of the AX‐CPT task structure. (b) Inhibitory task trial structure.

Each of the three blocks of trials was signalled with a presentation of an auditory cue lasting 3000 ms. This was followed by an on‐screen reminder of the rules displayed for 6000 ms (2 screens – each displayed for 3000 ms). Each trial started with a presentation of the cue for 500 ms and after a blank screen delay of 1200 ms, a probe was shown for a maximum of 5000 ms. After each response, a child was given verbal feedback: ‘Well done’ for correct responses, ‘Don't forget the rules’ for incorrect responses and ‘Too slow. Try to press the button faster next time’ – for missed responses. A schematic presentation of the AX‐CPT task structure is shown in Figure 1a and the trial structure in Figure 1b. Each block consisted of 30 trials presented randomly. Character‐fruit pairings and ‘like’ and ‘dislike’ rules were counterbalanced across participants.

Proactive and reactive d‐prime indices were calculated from the normalized hit rate on AX trials (the proportion of correct responses on AX trials) and false alarm rate on BX trials (the proportion of errors on BX trials): Z(H_AX_) – Z(F_BX_). For the d‐prime index of reactive control, a false alarm rate on AY trials was used: Z(H_AX_) – Z(F_AY_). To adjust for trials where zeros were present, a constant of 0.5 was added to all data cells (Macmillan & Creelman, 2005). Indices of proactive and reactive inhibitory control were highly correlated (*r* = .82, *p* < .001) and showed similar patterns of correlation with ADHD and conduct problems. Therefore, the mean of proactive and reactive indices was calculated to transform these into a single index of inhibitory control.

#### Temperament

Temperament was measured with the very short form of the Children's Behaviour Questionnaire (CBQ; Putnam & Rothbart, 2006). Parents rated their children's behaviour on a 7‐point scale ranging from ‘extremely untrue’ to ‘extremely true’ of their child. CBQ consists of three broad factors, each including 12 items: effortful control, negative affect and surgency. The effortful control factor encompasses fine‐grained traits such as attention, low‐intensity pleasure and inhibitory control. Example items included: ‘When drawing or colouring in a book, shows strong concentration’ or ‘Prepares for trips and outings by planning things s/he will need’. Higher scores on this factor reflect better effortful control. Cronbach's Alpha in the present sample was α = .60. Negative affect includes traits such as reactivity, fear, frustration, sadness and discomfort, with higher scores reflecting more negative affect. Example items included ‘Tends to become sad if the family's plans don't work out’ or ‘Is afraid of burglars or the boogie man’. In the present sample, Cronbach α = .66. Finally, surgency encompasses traits such as activity level, high‐intensity pleasure, positive anticipation and impulsivity. ‘Example items included: Likes going down high slides or other adventurous activities’ or ‘Is full of energy, even in the evening.’ In the present sample, Cronbach α = .67.

#### Psychopathology

Psychopathology was conceptualized dimensionally with ADHD symptoms and conduct problems, which were measured with the respective subscales of the Strengths and Difficulties Questionnaire completed by parents. It is a well‐validated questionnaire assessing psychopathology symptoms in 2‐17‐year‐old children (Goodman, 1997). The ADHD symptoms subscale consisted of five items, for example, ‘Constantly fidgeting or squirming’ or ‘Restless, overactive, cannot stay still for long’. The conduct problems subscale also consisted of five items, for example, ‘Often has temper tantrums or hot tempers’ or ‘Often fights with other children or bullies them’. Each item was rated on a 3‐point scale ranging from not true to certainly true and a higher score represented greater symptom severity.

### Analysis

Bivariate Pearson (for continuous variables) and point biserial (for dichotomous variable, sex) correlation coefficients were calculated for the associations between inhibitory control, negative affect, surgency, effortful control, ADHD symptoms and conduct problems, age and sex. Partial correlations controlling for conduct problems were also calculated. Path analysis based on 5000 bootstrap samples was conducted using R *lavaan* package (Rosseel, 2012). We did not conduct any formal a priori sample size calculations, as for path models, these are complex and require computationally intensive methods. However, recent research by Sim et al. (2021), who used a series of Monte Carlo simulations, suggests that for simple bootstrapped path models, such as the one used in our analysis, a minimum sample size of between 40–80 (depending on the effect size) participants should be sufficient.

Only variables that were significantly correlated with ADHD symptoms or inhibitory control were included in the next step of the analysis (path model), given the modest size of the sample. Therefore, negative affect and surgency were not included in further analysis. Direct and indirect paths were modelled from ADHD symptoms to inhibitory control via effortful control, and vice versa, using age and conduct problems as the covariates. Given the cross‐sectional nature of the data, the choice of predictor and outcome was arbitrary, and we expected the models with ADHD symptoms, and those with inhibitory control as the predictor and the outcome, respectively, to be roughly equivalent. We reported the model with ADHD symptoms as the predictor in the main body of the paper (Model 1) and the equivalent model with inhibitory control as the predictor in Appendix S1 (Model 2). The fit of the models was assessed by applying the ‘rules of thumb’, which indicate a good fit (Hu & Bentler, 1999), to the Comparative Fit Index (CFI >0.95) and the Root Mean Square Error of Approximation (RMSEA < 0.06). For the R code used in the analysis, see Appendix S1.

## RESULTS

On average, children completed 88 trials of the AX‐CPT. Responses made within 200 ms of probe presentation were treated as anticipatory (Conners & Staff, 2000) and excluded from the analyses, which removed 2.4% of the trials. Mean accuracy for trials requiring frequent target response (AXE) was 88.8% (*SD* = 17.5). For trials requiring rare non‐target response, accuracy on AY trials was 65.1% (*SD* = 29.4), on BX trials was 52.9% (*SD* = 31.1) and on BY trials was 62.6% (*SD* = 25.2).

A mean d‐prime index of proactive inhibition was 1.85 (*SD* = 1.26) and a mean d‐prime index of reactive inhibition was 2.24 (*SD* = 1.28). Correlations between both d‐prime indices, ADHD symptoms and conduct problems are shown in Table 2.

**TABLE 2:** Correlations between the individual indices of inhibitory control, ADHD symptoms and CP.

	ADHD symptoms	Conduct problems	Proactive inhibition
ADHD symptoms	–		
Conduct problems	.28*	–	
Proactive inhibition	−.20	−.36**	–
Reactive inhibition	−.26*	−.37**	.82**

*p < .05, **p < .01; all tests are two‐tailed.

Bold numbers indicate significant correlations.

The means, standard deviations (*SD*) and correlations between age, sex, index of inhibitory control, temperament variables and ADHD symptoms and partial correlations controlling for conduct problems are shown in Table 3.

**TABLE 3:** Means (*SD*) and correlations and partial correlations controlling for conduct problems (shaded area) between demographic, cognitive, temperament and ADHD symptoms variables.

	Mean (*SD*)	Age	Male	ADHD	Sur	NA	EC	IC
Age	5.5 (1.0)	1	−.01	−.01	−.07	.135	.01	.42**
Male sex	–	−.09	1	.17	.38**	−.04	−.20	−.14
ADHD symptoms (ADHD)	3.9 (2.2)	.00	.12	1	.12	−.03	−.40**	−.15
Surgency (Sur)	4.7 (0.8)	−.07	.35**	.15	1	−.37**	−.21	−.04
Negative affect (NA)	4.2 (0.9)	.13	−.08	.05	−.31**	1	.05	−.01
Effortful control (EC)	5.6 (0.6)	.01	−.18	−.42**	−.23*	.01	1	.27*
Inhibitory control (IC)	2.0 (1.2)	.39**	−.07	−.24*	−.09	−.12	.30**	1

*p < .05, **p < .01; all tests are two‐tailed.

Bold numbers indicate significant correlations.

Shaded area shows partial correlations controlling for conduct problems.

Symptoms of ADHD were significantly negatively correlated with effortful and inhibitory control (*r* = −.42, *p* < .001 and *r* = −24, *p* = .033, respectively). After controlling for conduct problems, only the association with effortful control remained significant (*r* = −.40, *p* < .001). Inhibitory control was significantly positively correlated with effortful control and age (*r* = .30, *p* = .009 and *r* = .39, *p* < .001, respectively). These correlations remained significant after controlling for conduct problems. Surgency was positively correlated with male sex (*r* = .35, *p* = .002) and negatively with negative affect (*r* = −.31, *p* = 006) and these associations remained significant after conduct problems were controlled for. No other correlations were significant (all *p*s > .05). Given that neither negative affect nor surgency were significantly correlated with ADHD symptoms or inhibitory control, these were not included in the next analysis step.

**FIGURE 2:**
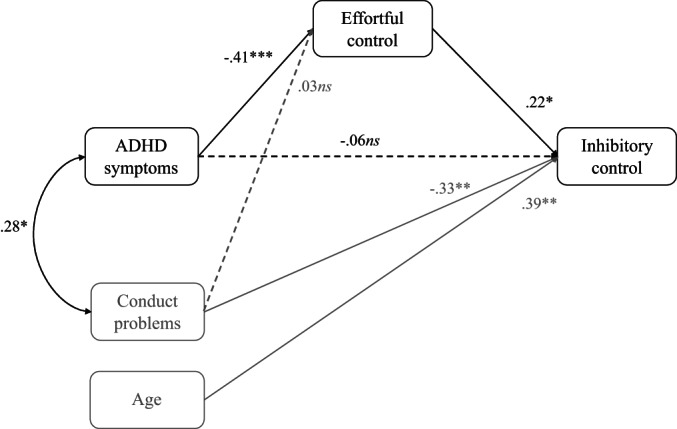
MODEL1: A path MODEL of the associations between ADHD symptoms, effortful control and inhibitory control. Asterisks denote: *p < .05, **p < .01, ***p < .001. Solid lines represent statistically significant associations and dashed lines associations that are not statistically significant. Grey colour depicts a covariate (age and conduct problems).

Both path models achieved a very good fit, CFI = 1.0, RMSEA = .00, *χ* ^2^(10) = 54.7, *p* < .001. In Model 1, there was a significant negative path from ADHD symptoms to effortful control (Figure 2; *β* = −.41, *p* < .001). There was also a significant indirect path from ADHD symptoms to inhibitory control via reduced effortful control (*β* = −.09, *p* = .043; see Appendix S1 for analysis with inhibitory control as the predictor and ADHD symptoms as the outcome). However, there was no direct path from ADHD symptoms to inhibitory control (*β* = −.06, *p* = .603; see Appendix S1 for analysis with inhibitory control as the predictor). Further, the model showed a significant direct negative path from conduct problems to inhibitory control (*β* = −.33, *p* = .005), but the path to effortful control was not significant (*β* = −.03, *p* = .788). Finally, inhibitory control was also positively associated with effortful control (*β* = .22, *p* = .030) and with age (*β* = .39, *p* < .001).

## DISCUSSION

In this cross‐sectional study of children recruited from the community, we examined the relationship between ADHD symptoms, inhibitory control deficits and effortful control. We tested the specific hypothesis that the association between ADHD symptoms and inhibitory control deficits is grounded in temperamental variations in effortful control. To explore the specificity of these relationships we also examined two other temperament factors, namely, negative affect and surgency, while also controlling for conduct problems.

The results of bivariate correlations and path analysis showed that ADHD symptoms and conduct problems were associated with deficits in inhibitory control measured by the AX‐CPT task. This is consistent with the previous meta‐analytical results, which showed that inhibitory deficits are not a unique characteristic of a particular disorder, for example, ADHD, but are found across many childhood and adult psychopathologies (Lipszyc & Schachar, 2010; Oosterlaan et al., 1998; Wright et al., 2014). However, there was no evidence that ADHD symptoms were associated with higher negative affect or greater levels of surgency.

While the lack of association between ADHD symptoms and negative affect is somewhat surprising, given the previous research findings (De Pauw & Mervielde, 2011; Healey et al., 2011; Martel et al., 2014), it may be explained by the severity of ADHD symptoms in children that participated in these studies. Our study included a community sample of children who did not, on average, have elevated ADHD scores measured with the SDQ. In contrast, the studies that showed increased levels of negative affect in ADHD included children with a clinical diagnosis or, at least, children who were specifically recruited because of their ADHD‐related difficulties. Regarding surgency, our findings add to the body of research providing evidence that ADHD symptoms are not linked to increased surgency (De Pauw & Mervielde, 2011) despite their associations with increased activity level (Kostyrka‐Allchorne et al., 2020) – a trait included in this factor. However, given the heterogeneity of the traits that comprise surgency (e.g., high‐intensity pleasure, positive anticipation, impulsivity/sociability and activity level), it may be difficult to draw clear conclusions about the role of this temperament factor.

Importantly, the results of partial correlations and path analysis also showed the specificity of the associations between inhibitory deficits and ADHD symptoms and conduct problems. In this regard, there were two specific findings of note.

First, when conduct problems were controlled for, the bivariate correlation between symptoms of ADHD and inhibitory performance reduced in effect size (*r* = −.24 to −.15). Although this reduction does not appear substantial in size, the correlation was no longer statistically significant. This finding contrasts with the results of Krieger et al. (2019), which showed that the association between ADHD and inhibitory performance persisted after controlling for co‐existing conduct disorder. However, the latter study included adolescents, who met the criteria for a clinical diagnosis of ADHD and measured inhibitory control as part of a wider executive function assessment, which may explain the different findings.

Second, the exploratory path analysis showed that the associations between ADHD symptoms, conduct problems and poor inhibitory task performance were unique to the type of psychopathology. Low effortful control fully explained the association between ADHD symptoms and poor inhibitory task performance. This finding fits with the cognitive‐energetic model first proposed by Sanders (1983) and subsequently extended to ADHD by Sergeant (2000, 2005) and Sergeant et al. (1999). In this model, ADHD deficits are the result of an interplay of top‐down and bottom‐up processes, which include basic cognitive processing such as motor response with effort and motivation needed to control arousal and complex cognition that comes under the umbrella of executive function (Sonuga‐Barke et al., 2010). In the context of our study, it is possible that children with high levels of ADHD symptoms struggled to maintain optimal arousal levels during a task that was boring and monotonous because of their dispositional regulatory difficulties. More broadly, our findings provide further evidence that focusing on a single underlying core deficit (e.g., Barkley, 1997) cannot adequately account for the complexity of ADHD‐related difficulties (Pennington, 2005; Sonuga‐Barke, 2005). Instead, models that incorporate multiple independent pathways from deficits in top‐down cognitive abilities and variations in effort and emotion regulation to ADHD (Sergeant et al., 1999; Sonuga‐Barke, 2002) might be better suited to explain heterogeneity that characterizes this disorder.

In contrast to the *indirect* association seen in relation to the inhibitory control deficits and ADHD symptoms, exploratory analysis of the associations between conduct problems and inhibitory control showed a *direct* negative link between these variables. This fits with the overall pattern of previous findings showing the associations between inhibitory deficits in early childhood and general externalizing difficulties (Schoemaker et al., 2013; Utendale et al., 2011), aggression (Floyd & Kirby, 2001; Raaijmakers et al., 2008; Suurland et al., 2016) and ‘hard‐to‐manage’ behaviour (Hughes et al., 1998). What is less clear is the mechanism that underpins these associations. Considering that the tendency to violate rules is a feature of conduct problems, it is possible that poor inhibitory performance was driven by a deliberate lack of task engagement or not following the rules explained by the experimenter rather than the inability to overcome a pre‐potent response. This possibility should be explored in future research.

In addition to the above findings, our data showed that proactive and reactive inhibitory control were best modelled as a single construct. They were both highly correlated with each other and showed similar patterns of correlation with ADHD symptoms and conduct problems. This contrasts with the evidence for the two modes of inhibitory control, which are dissociable in early childhood. Prior research with children aged 8 years and younger provided evidence for a developmental shift from reactive inhibition to proactive inhibition (Chatham et al., 2009), which most likely occurs around the age of 5 to 6 years (Blackwell & Munakata, 2014; Gonthier et al., 2019; Lucenet & Blaye, 2014).

The evidence of the shift from automatic, stimulus‐driven to purposeful and goal‐driven behaviour fits with the generally accepted dual‐process approach, which characterizes much of the cognitive and developmental theory and research. However, this approach has been criticized for its lack of ability to account for the complexity and dynamic nature of human behaviour, which is likely to be underpinned by a combination of automatic and goal‐directed processes (Hommel & Wiers, 2017; Mekern et al., 2019). Our data are more consistent with a unitary approach proposed by Hommel and Wiers (2017). This model suggests that all behaviours are goal‐directed; however, depending on the circumstances, individuals choose actions that are simple and fast (i.e., automatic) or slow and controlled (intentional).

An important contribution of this study is that it brings together temperamental and child psychopathology symptoms to examine their contribution to inhibitory performance. Moreover, it differentiates between the nature of inhibitory deficits in ADHD symptoms and conduct problems – as revealed in their associations with effortful control. However, findings should be considered in the context of three limitations. First, while the cross‐sectional design of the study allowed us to take the first step in examining the potential role of temperament in the ADHD‐inhibitory control deficit links, we were unable to establish the developmental sequence or whether the observed associations persisted over time. One hypothesis that can be tested with longitudinal data, including that collected during the early pre‐school period, is whether alterations in primary inhibitory control deficits specifically and executive functions more generally represent an early neurodevelopmental pre‐cursor of the later development of ADHD that may be linked to temperamental differences in effortful control (Sonuga‐Barke & Halperin, 2010). Second, we measured inhibitory control with a single paradigm; multiple measurements of this construct using several tasks may provide a more robust estimate of inhibitory ability in young children. In addition, using an age‐appropriate version of the AX‐CPT task helped to ensure that children understood the rules and engaged with it. However, this has also limited our ability to make direct comparisons with other studies, which used an unmodified version of this task. Finally, while our sample included ethnically diverse children, the majority of parents were well‐educated. This could be due to the recruitment methods used, which relied on convenience sampling. Moreover, ADHD symptoms and conduct problems in the present non‐clinical sample of children were relatively low. Therefore, future research should attempt to recruit families representing a more diverse socio‐economic background and oversample children with more severe difficulties.

In conclusion, this study provides evidence that the association between ADHD symptoms and inhibitory control problems was statistically mediated by low effortful control while conduct problems were directly linked to poor inhibitory performance. Further, there was no evidence that ADHD symptoms in a community sample of children were linked to increased negative affect and higher levels of surgency. It is important for future research to include children with a wider range of ADHD‐ and conduct problem‐related difficulties, including clinically referred children.

## AUTHOR CONTRIBUTIONS

**Katarzyna Kostyrka‐Allchorne:** Formal analysis; project administration; supervision; writing – original draft. **Sam V. Wass:** Conceptualization; supervision; writing – review and editing. **Hodo Yusuf:** Investigation; writing – review and editing. **Vidya Rao:** Data curation; writing – review and editing. **Chloe Bertini:** Investigation; writing – review and editing. **Edmund J. S. Sonuga‐Barke:** Conceptualization; methodology; supervision; writing – review and editing.

## CONFLICT OF INTEREST

All authors declare no conflict of interest.

## Supporting information


Appendix S1

## Data Availability

The data that support the findings of this study are available from the corresponding author upon reasonable request.
