# Supplementary material for: Inhibitory deficits and symptoms of attention‐deficit hyperactivity disorder: How are they related to effortful control?
Source: Br J Dev Psychol. 2022 Sep 20;41(1):50–65. doi: 10.1111/bjdp.12432 (PMC10087402; doi:10.1111/bjdp.12432)
Supplement: Supplementary file 1 — Appendix S1 [file BJDP-41-50-s001.docx]

**Supplementary results: MODEL 2**

In MODEL 2 inhibitory control was entered as a ‘predictor’ variable and ADHD symptoms as an ‘outcome’ variable (Figure S1).

*Figure S1. MODEL2: Path model of the associations between inhibitory control, effortful control and ADHD symptoms. Asterisks denote: *p < .05, **p <.01, ***p<.001. Solid lines represent statistically significant associations and dashed lines associations that are not statistically significant. Grey colour depicts a covariate (age and conduct problems).*

Model 2 achieved a very good fit, CFI = 1.0, RMSEA=.00, χ2(10) = 54.7, p < .001. In Model 2, there was a significant positive path from inhibitory control to effortful control (Figure S1; β = .28, p =.020). There was also a significant indirect path from inhibitory control to ADHD symptoms via reduced effortful control (β = -.10, p = .049). However, there was no direct path from inhibitory control to ADHD symptoms (β = -.05, p = .644). Further, the model showed that inhibitory control was significantly negatively associated with conduct problems (β = -.38, p = .001) and positively with age (β= .39, p < .001). Finally, effortful control was significantly negatively associated with ADHD symptoms (β= -.37, p = .001), but the path from conduct problems to effortful control was not significant (β = -.04, p = .747).

**SDQ hyperactivity scores distribution**

Figure S2 presents a distribution of the SDQ hyperactivity scores in the normative sample of school aged children 4-15 (source: <https://www.sdqinfo.org/norms/UKNorms.html>) and in the present study sample.

0 1 2 3 4 5 6 7 8 9 10

*Figure S2. Distribution of the SDQ hyperactivity scores in the normative and the current study sample.*

**R script used in the analysis**

library(lavaan)

library(lavaanPlot)

library(psych)

##### Read in data ######

dat <- read.csv('path.csv')

##### Path analysis with lavaan package #####

#####MODEL 1: ADHD as independent variable and IC as outcome#####

model.adhd_cp <- 'cp ~ ~ adhd

                  inhCtrl ~ b1*effCtrl +c1*adhd + c2*cp + c3*age

                  effCtrl ~ a1*adhd

                  effCtrl ~ a2*cp

                  indirect1 := a1*b1

                  indirect2 :=a2*b1

                  direct1 := c1

                  direct2 := c2

                  direct3 := c3

                  total1 := c1+ (a1*b1)

                  total2 :=c2 + (a2*b1)'

fit <- sem(model.adhd_cp, data = dat, se = 'bootstrap', bootstrap = 5000,)

summary(fit, fit.measures = TRUE, standardized=T, rsquare=T)

##### Create a figure (N.B.: the figure included in the manuscript was drawn manually) ######################################################################

labels <- list(adhd = "ADHD", cp = "CP", effCtrl = "Effortful control",

               age = "Age", inhCtrl = "Inhibitory control")

lavaanPlot(model = fit, labels = labels, node_options = list(shape = "box", fontname = "Helvetica"),

           edge_options = list(color = "black"), coefs = T, stand = T, sig = 0.05,

           covs = T, stars = c('covs','regress'))

#####MODEL 2: IC as independent variable and ADHD as outcome#####

model.ic_cp <- ' cp ~ ~ inhCtrl

                  age ~ ~ inhCtrl

                  adhd ~ b1*effCtrl +c1*inhCtrl + c2*cp

                  effCtrl ~ a1*inhCtrl

                  effCtrl ~ a2*cp

                  indirect1 := a1*b1

                  indirect2 :=a2*b1

                  direct1 := c1

                  direct2 := c2

                  total1 := c1+ (a1*b1)

                  total2 :=c2 + (a2*b1)'

fit <- sem(model.ic_cp, data = dat, se = 'bootstrap', bootstrap = 5000,)

summary(fit, fit.measures = TRUE, standardized=T,rsquare=T)

##### Create a figure (N.B.: the figure included in the manuscript was drawn manually) ### #########################################################################

labels <- list(adhd = "ADHD", cp = "CP", effCtrl = "Effortful control",

               age = "Age", inhCtrl = "Inhibitory control")

lavaanPlot(model = fit, labels = labels, node_options = list(shape = "box", fontname = "Helvetica"),

           edge_options = list(color = "black"), coefs = T, stand = T, sig = 0.05,

           covs = T, stars = c('covs','regress'))
